# *Tm-1* back in business: an allele from *Solanum pennellii* accessions plays a major role in ToBRFV resistance

**DOI:** 10.1007/s00122-025-05036-1

**Published:** 2025-09-12

**Authors:** Romanos Zois, Mireille van Damme, Martin Verbeek, Luuk D. H. Veenendaal, Yuling Bai, Anne-Marie A. Wolters

**Affiliations:** 1 Plant Breeding, Wageningen University & Research, Droevendaalsesteeg 1, 6708 PB Wageningen, The Netherlands; 2 Graduate School Experimental Plant Sciences, Wageningen University & Research, Droevendaalsesteeg 1, 6708 PB Wageningen, The Netherlands; 3 Biointeractions & Plant Health, Wageningen University & Research, Droevendaalsesteeg 1, 6708 PB Wageningen, The Netherlands

## Abstract

**Key message:**

The *Tm-1* allele from *Solanum pennellii* accessions together with an additional, likely recessive, locus are required for complete ToBRFV resistance.

**Abstract:**

The *Tobamovirus* Tomato Brown Rugose Fruit Virus (ToBRFV) poses a significant threat to global tomato production. ToBRFV is a mechanically transmitted virus containing a single-stranded positive sense RNA genome. Disease symptoms include brown, rough patches on fruit surfaces, leaf mosaicism and shape abnormalities, and, in advanced stages, total collapse of infected plants. ToBRFV was first detected in the Middle East in 2014 and has rapidly spread to multiple countries across Asia, Europe, and America. In recent years, numerous studies have focused on the identification of ToBRFV resistance traits that are suitable for tomato breeding programs. In this study, we identified five ToBRFV-resistant accessions of *Solanum pennellii*, a wild relative of cultivated tomato. We confirmed that the major gene controlling this resistance trait is the *S. pennellii* allele of *Tm-1*. *Tm-1* was previously identified in *S. habrochaites* as a semidominant Tomato Mosaic Virus (ToMV) resistance gene. Our results show that full resistance to ToBRFV disease requires an additional undescribed locus. These results show the potential of *S. pennellii* as a novel source of resistance against ToBRFV.

**Supplementary Information:**

The online version contains supplementary material available at 10.1007/s00122-025-05036-1.

## Introduction

Tomato (*Solanum lycopersicum*) is among the most widely cultivated vegetable crops globally, playing a critical role in both commercial agriculture and local economies (FAO 2022). However, tomato production is frequently threatened by viral diseases, which can lead to devastating losses (Rivarez et al. 2021). International trade and movement of people, along with the widespread use of single resistance genes in large-scale monocultures, accelerate the spread and evolution of viruses (Jones 2021). Among these threats, Tomato brown rugose fruit virus (ToBRFV) has emerged as one of the most severe, posing significant challenges to growers worldwide (van Damme et al. 2023).

First reported in 2014, ToBRFV belongs to the *Tobamovirus* genus and has rapidly spread across major tomato-growing regions, including Europe, the Middle East, and the Americas (Zhang et al. 2022). The virus is highly contagious and spreads mechanically via infected tools, human contact, insect pollinators, contaminated soil, and plant material (Caruso et al. 2022). ToBRFV induces severe symptoms like leaf mosaic patterns, yellowing, and wrinkled (rugose) patches on the fruit (González-Concha et al. 2023) which reduces the marketable yield. Although agronomic and hygiene measures can help prevent spreading, no chemical treatments can cure infected plants, making resistance breeding the most sustainable and efficient control strategy (Gómez et al. 2009; Jones 2006).

Historically, tomato resistance to tobamoviruses has relied on well-characterized genes like *Tm-1* and *Tm-2*/*Tm-2*^*2*^, which confer protection against strains of tobacco mosaic virus (TMV) and tomato mosaic virus (ToMV) (Hall 1980; Ishibashi et al. 2007). *Tm-2* and its allelic variant *Tm-2*^*2*^ are dominant resistance genes encoding coiled-coil, nucleotide-binding leucine-rich repeat (CC-NLR) proteins. These proteins recognize the TMV and ToMV movement protein, triggering a hypersensitive response (HR) to halt infection. Found in *S. peruvianum* accessions and located on chromosome 9, *Tm-2*^*2*^ is preferred in breeding programs due to its durability over *Tm-2* (Lanfermeijer et al. 2003; Meshi et al. 1989; Strasser and Pfitzner 2007; Weber et al. 2004).

*Tm-1* is a semidominant gene introgressed from *S. habrochaites* PI126445 (Pelham 1966) that encodes a 754-amino acid (aa) protein. Tm-1 inhibits tobamovirus replication by binding to the helicase domain of the viral replication protein. This binding restricts the ability of the viral replicase to interact with host factors that facilitate viral replication (Ishibashi 2010; Ishibashi et al. 2012; Ishibashi and Ishikawa 2014). The Tm-1 protein contains two conserved domains: an uncharacterized N-terminal domain and a C-terminal TIM barrel-like domain (Ishibashi et al. 2007). Only the N-terminal domain is necessary for Tm-1’s inhibitory activity, as a C-terminal-truncated Tm-1 protein was still able to inhibit viral replication (Ishibashi et al. 2014; Kato et al. 2013). Furthermore, in *S. habrochaites* accessions a small region of the Tm-1 N-terminal domain has been found to be positively selected in response to TMV strains able to overcome the initial *Tm-1* based resistance, underscoring its importance in the coevolution of tobamoviruses and *Solanum* species (Ishibashi et al. 2012). Interestingly, different allelic variants of *Tm-1* show differential resistance responses against various tobamoviruses (Ishibashi et al. 2009). For instance, the *S. lycopersicum tm-1* allele is not functional against ToMV and TMV but can inhibit the replication of other tobamoviruses, such as Tobacco Mild Green Mosaic Virus (TMGMV) and Pepper Mild Mottle Virus (PMMoV). Additionally, the functionality of Tm-1 against TMV has been shown to be temperature-dependent, exhibiting effective resistance at or below 25 °C while losing its effectiveness at 33 °C and above (Fraser and Loughlin 1982).

When ToBRFV emerged, all commercial tomato varieties were susceptible, even those that were resistant to other tobamoviruses such as ToMV, with the primary resistance gene being *Tm-2/Tm-2*^*2*^ (Hak and Spiegelman 2021; Jaiswal et al. 2024; Luria et al. 2018; Zinger et al. 2021). This has propelled the search for novel resistance traits that can withstand ToBRFV infection. Although initially *Tm-1* and *Tm-2* have been reported as ineffective against ToBRFV, recent studies indicate that they can play a role in novel resistance traits against the virus. Specifically, the *Tm-1* locus from certain tomato cultivars, when combined with loci on chromosome 11 and/or chromosome 9, provides resistance (Ashkenazi et al. 2020; Zinger et al. 2021, 2025). Regarding *Tm-2*, specific artificial amino acid changes can lead to a gain of function providing resistance against ToBRFV (Lindbo 2022). However, no naturally occurring variants of *Tm-2* have been reported to confer resistance to ToBRFV.

Recent research has highlighted wild *Solanum* species as valuable sources of resistance to ToBRFV. Several accessions from *S. pimpinellifolium*, *S. chilense*, *S. corneliomulleri*, *S. habrochaites*, *S. peruvianum*, and *S. ochranthum* have shown resistance to ToBRFV (Jaiswal et al. 2024; Jewehan et al. 2022a, 2022b; Kabas et al. 2022; Topcu et al. 2025). These findings emphasize the potential of diverse germplasm to contribute to the development of cultivars with durable, multilayered resistance. Notably, a resistance trait conferred by a dominant NBS-LRR gene on chromosome 8 has been introgressed from *S. habrochaites* (Ykema et al. 2020). However, ToBRFV isolates capable of overcoming this resistance have already emerged (Zisi et al. 2024).

In our study, we identified robust resistance in several *S. pennellii* accessions and confirmed the essential role of the *Tm-1* allele in this resistance trait. We also pinpointed the nine most relevant Tm-1 amino acids involved in ToBRFV resistance. Furthermore, we demonstrated that full resistance requires an additional locus from the resistant *S. pennellii* accessions, which is likely inherited recessively and distinct from those reported in other studies (Ashkenazi et al. 2020; Zinger et al. 2021, 2025). These findings provide valuable insights for breeders aiming to mitigate the impact of ToBRFV and offer tomato growers a sustainable solution to this viral threat.

## Materials and methods

### Plant material

Ten accessions of *S. pennellii*, LA 0716, LA 0750, G1.1559, G1.1608, G1.1610, LA 1356, LA 1656, LA 1724, LA 2177, and LA 2580 and the *S. lycopersicum* cv. Moneymaker (MM) were used in this study (in-house collection of Plant Breeding, Wageningen University and Research (WUR)). F1 plants were obtained from ToBRFV disease-resistant individuals from accessions LA 0716 and LA 0750 that were crossed with susceptible MM. Individual F1 plants were selfed to obtain F2 populations, F2 (LA 0716) and F2 (LA 0750).

### Plant growth conditions

For germination, the seeds were soaked in half-strength household bleach (ca. 2.7% sodium hypochlorite) for 30–60 min, then rinsed in running water for several minutes. Subsequently, the seeds were put on Whatman® cellulose filter paper in petri dishes containing 1/2 × Murashige and Skoog Basal Medium powder (MS) medium without sucrose. The petri dishes were placed in dark at 25 °C for two days. Then, the petri dishes were moved into a growing chamber with artificial light and 25 °C until the seedlings reached cotyledon stage. The seedlings were transferred into individual pots and moved into the quarantine greenhouse compartment free of pathogens and insects. The growing conditions were stable and controlled throughout the experiments (21°C/19°C (day/night) with 60% relative humidity and day length of 16 h with the light power of 250 W.

### ToBRFV inoculation

A Dutch isolate of tomato brown rugose fruit virus (ToBRFV-NVWA, genus *Tobamovirus*, species *Tobamovirus fructirugosum*; NVWA 33610411, NCBI accession code MN882011) was propagated in tomato cv. Moneymaker. Inoculum was prepared by grinding infected tomato leaves displaying clear ToBRFV symptoms in 0.03 M Na–K-phosphate buffer (pH 7.7). The optimal inoculum dilution (1:40–1:50) in inoculation buffer was determined through empirical testing using serial dilutions in *Nicotiana glutinosa*. Plant inoculation was conducted by dusting cotyledons and the first true leaves with Carborundum (500 mesh) and gently rubbing the leaves with gloved fingers dipped in the inoculum. The climate was set at a 20% relative humidity, a 16/8 h day night cycle at 20 °C/18 °C (day/night) regime throughout the disease assay.

### Phenotyping: disease severity index (DSI) and ToBRFV test

**Fig. 1:**
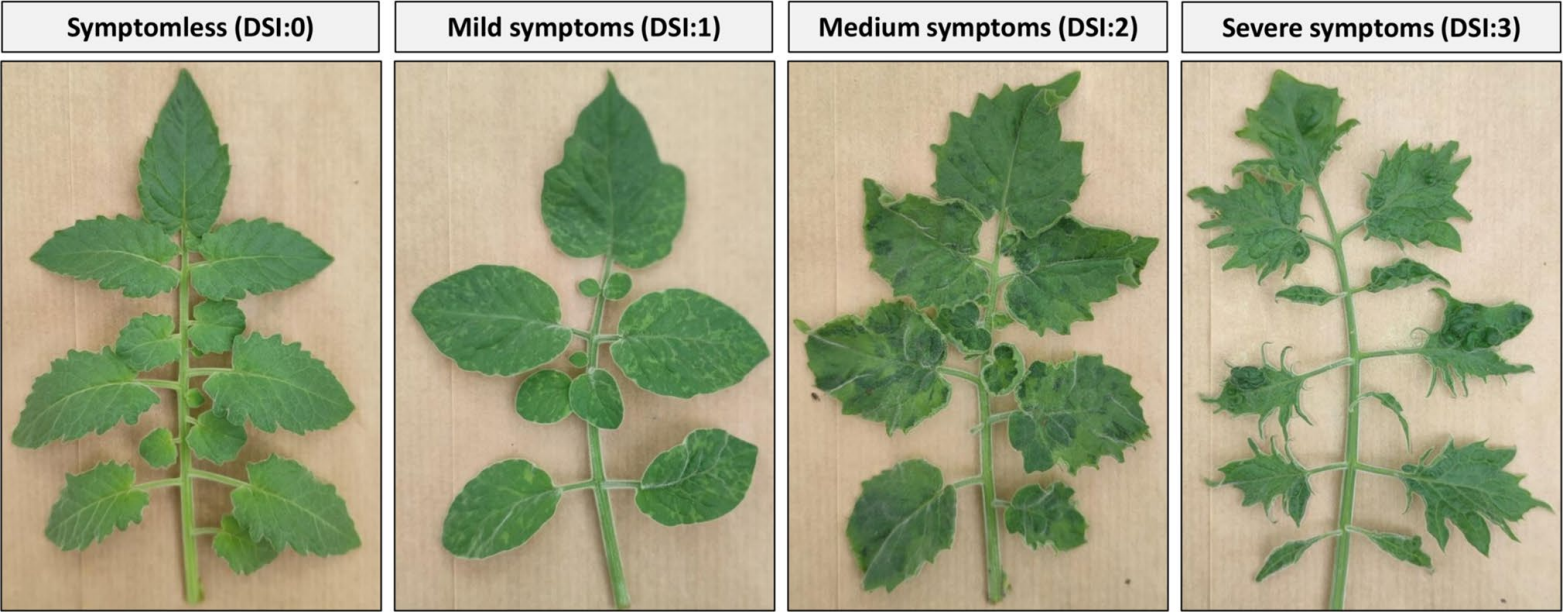
Disease severity index (DSI) used for ToBRFV symptoms observed in the F2(LA 0716) population ranging from DSI 0 (no symptoms) to DSI 3 severe symptoms

Symptoms were assessed at 3 to 4 weeks post-ToBRFV inoculation. A disease severity index (DSI) was developed and used for scoring F2 generations derived from crosses of *S. pennellii* accessions with MM plants (Fig. 1). The DSI ranged from 0 to 3, where 0 is scored as asymptomatic, 1 for mild symptoms of leaf mosaic, 2 for medium symptoms with strong leaf mosaicism, mild leaf abnormalities and wrinkling, and 3 for severe symptoms of leaf mosaicism, abnormalities, and wrinkling. A commercial ToBRFV ImmunoStrip® assay from Agdia was used to determine the systemic spread and presence of ToBRFV, based on ToBRFV coat protein detection, in uninoculated plant parts. Detection of ToBRFV with the ImmunoStrip assay was scored with a (+), while if ToBRFV was not detected a (-) score was given.

### Quantification of ToBRFV Coat protein levels

Stain-free protein gel assays were used to quantify the presence of ToBRFV coat protein in leaf samples. Proteins were extracted from single leaflets in Bioreba bags with 2 ml protein extraction buffer (SEB1 from Agdia). 30 μl of extracted protein was mixed with 10 μl of 4 × Laemmli buffer with beta-mercaptoethanol (β-ME). The mixture was heated for five minutes at 95°C. 10 μl of the samples were loaded in Mini-PROTEAN TGX Stain-Free precast gels from BIO-RAD. Gel pictures were taken with GelDoc Go Gel Imaging System Bio-Rad.

### Development of cleaved amplified polymorphic sequence (CAPS) markers

CAPS markers were used for the genetic analysis of important tobamovirus resistance loci. The primers for the CAPS markers (Table 1) were designed based on the tomato Heinz genome (SL4.0 version) and the *S. pennellii* LA 0716 genome (Bolger et al. 2014) retrieved from Sol genomics database (https://solgenomics.net; for *Tm-1*: *S. lycopersicum Solyc02g062560, S. pennellii Sopen02g013570*, and for *Tm-2*: *S. lycopersicum Solyc09g018220*, *S. pennellii Sopen09g035210*). The primers for the QTL11 marker were designed to amplify a region on chromosome 11 upstream of Solyc11g018660, while the primers for the QTL9 marker target a region on chromosome 9 (within Solyc09g065470), as described by Zinger et al. (2021) and Ashkenazi et al. (2020), respectively. Genomic DNA was extracted from the youngest leaf of each F2 individual using a modified cetyltrimethylammonium bromide (CTAB) protocol as described by Schenk et al. (2023). These genomic DNA samples were used as template for PCRs with the CAPS marker primers. The PCR products were digested with appropriate restriction enzymes (Table 1). The products of digestion were separated on a 1.5% agarose gel and visualized with GelDoc Go Gel Imaging System by Bio-Rad.

**Table 1:** PCR primers and conditions for CAPS markers and the restriction enzymes used

Name	Primer sequence (5'→ 3')	Chromosomal location	PCR product size	Restriction enzyme	Digestion products
*Tm-1* marker	Fw: TCTCACCATTCTCACACTGAGTTAC	Spenn-ch02: 36,968,554–36,967,408	*S. pennellii:* 1147 bp	*BamHI*	*S. pennellii:* ~ 500, 650 bp
	Rv: ACTGAAGGAAACAATACCAAGTCTG	SL4.0ch02: 32,289,329–32,290,472	*S. lycopersicum:* 1144 bp		*S. lycopersicum:* ~ 430, 720 bp
*Tm-2* marker	Fw: CCTTTTTCATTAATGTGCAGCTGCC	Spenn-ch09: 18,228,467–18,227,445	*S. pennellii:* 1023 bp	*EcoRV*	*S. pennellii:* ~ 1023 bp
	Rv: GAGACGTGATTATCATTCTACTGCCG	SL4.0ch09: 13,658,610–3659650	*S. lycopersicum:* 1041 bp		*S. lycopersicum:* ~ 297, 744 bp
QTL11 marker	Fw: GGTACCCTCTCAATCTCAAGGTC	Spenn-ch11: 9,645,767–9,646,451	*S. pennellii:* 685 bp	*TaqI*	*S. pennellii:* ~ 230, 450 bp
	Rv: GAATTTACACGCCACCTTCCTC	SL4.0ch11: 8,971,865–8,972,550	*S. lycopersicum:* 686 bp		*S. lycopersicum:* ~ 190, 490 bp
QTL9 marker	Fw: TTCTTCCTTTGCCTGTTCTATTTG	Spenn-ch9: 74,872,135–74,872,729	*S. pennellii:* 649 bp	*TaqI*	*S. pennellii:* ~ 170, 480 bp
	Rv: GACTCATTACATTGTTCCTCCC	SL4.0ch9: 59,672,886–59,673,482	*S. lycopersicum:* 597 bp		*S. lycopersicum:* ~ 170, 420 bp

### Statistical analysis

Chi-squared (χ^2^) tests of independence were conducted in Microsoft Excel to assess the association between CAPS genetic markers and phenotypic results based on the disease severity index (DSI). The analysis evaluated whether the genotypic distribution of individuals in the F₂ (LA 0716) population differed significantly across phenotypic groups.

### Virus-induced gene silencing (VIGS)

The target sequences for silencing *S. pennellii Tm-1* and *Tm-2* alleles were selected using the Sol genomics VIGS tool (Fernandez-Pozo et al. 2015). The selected target regions for *S. pennellii* and *S. lycopersicum Tm-1* alleles (Solyc02g062560 and Sopen02g013570) and *Tm-2* alleles (Solyc09g018220 and Sopen09g035210) shared high levels of sequence homology*,* and therefore, the same construct can be used to silence the respective *Tm* genes in both *Solanum* species. Amplified PCR fragments of the target region (300 bp) of *Tm-1* and *Tm-2* genes were directionally cloned in pENTR™ by TOPO® Cloning strategy. The *Tm-1* fragment was amplified with primers *Tm-1_*VIGS_Fw (5’-GTAGGAGTGACAGTTGTTGATGTC-3’) and *Tm-1_*VIGS_Rv (5’-AACTTTTGGGATTCCAATTGGAAG-3’), while the *Tm-2* fragment was amplified with primers *Tm-2_*VIGS_Fw (5’-TAGAAGGGTTGTTGACATTGACCGA-3’) and *Tm-2_*VIGS_Rv (5’-GAAACGTAGACCAGTCCAGAACACT-3’). *S. pennellii* (LA 0716) cDNA was used as template, and a high fidelity Taq polymerase (Phusion) was used as enzyme. Correctness of the sequence was confirmed by Sanger sequencing of the plasmids. The target regions were cloned into the TRV2 vector (Liu et al. 2002) by using Gateway cloning strategy. TRV2 vectors with *Tm-1* and *Tm-2* targeting regions were transformed into *Agrobacterium tumefaciens* strain GV3101. As a negative control, a TRV2 vector carrying a 396-bp fragment of the *β-glucuronidase* (*GUS*) gene, which has no homology with endogenous *Solanum* genes, was used. Agrobacterium cells carrying the constructs were grown overnight in LB media with appropriate antibiotics. When OD_600_ reached ~ 0.7, the cultures were centrifuged. The pellets were diluted in infiltration buffer (pH 5.7) containing 200 µM acetosyringone, 10 mM 2-(N-morpholino) ethane sulfonic acid (MES) and 10 mM MgCl2, and the OD_600_ of each construct was adjusted to 2. TRV1 and TRV2 cultures were mixed in 1:1 ratio resulting in OD_600_ equal to 1 of each. Before inoculation, Silwet L-77 (0.02%) was added to the inoculum. Seedlings were submerged in the *Agrobacterium* suspension and both surfaces of the cotyledons, as well as the hypocotyl and the roots of the seedlings were brush-inoculated (Cox et al. 2019). Agro-brush-inoculation was performed on 10–15-day-old seedlings (cotyledon stage) by using sterilized paint brushes. Inoculated plants were individually transplanted into plastic pots with potting soil. Two weeks after TRV agro-brush-inoculation the plants were mechanically inoculated with ToBRFV.

### Cloning and sequencing of *Tm-1* alleles

Young leaf samples were collected from all tested *S. pennellii* accessions and cv. MM plants. Total RNA was extracted using the RNeasy Mini Kit (QIAGEN), and RNA concentrations were measured with a NanoDrop™ One Spectrophotometer (Thermo Fisher Scientific). cDNA synthesis was performed using SuperScript™ III Reverse Transcriptase (Thermo Fisher Scientific) following the manufacturer's protocol.

*Tm-1* primers were designed based on the tomato Heinz genome (SL4.0 version) and the *S. pennellii* LA 0716 genome retrieved from the Sol Genomics database (Bolger et al. 2014). The primer set amplifies *Tm-1* alleles from both cv. MM and *S. pennellii* accessions. The forward primer (*Tm-1_*Fw: 5’-ATGGCAACTGCACAGAGT-3’) begins from the start codon, while the reverse primer (*Tm-1_*Rv: 5’-TCACTCCATAGATATAGACTTGTAC-3’) starts from the stop codon. PCR was performed using cDNA from the different *S. pennellii* accessions and cv. MM as templates, *Tm-1* primers, and Phusion High-Fidelity DNA Polymerase (Thermo Fisher Scientific). PCR products were cloned into a vector using the Zero Blunt™ PCR Cloning Kit (Thermo Fisher Scientific), and subsequently transformed into *E. coli* Top10 cells. Transformed cells were selected, and the correctness of their plasmids was confirmed by PCR and Sanger sequencing. Plasmids carrying the *Tm-1* allele from each *S. pennellii* accession and cv. MM were further analyzed by whole-plasmid sequencing. The amino acid sequences of Tm-1 variants were compared using the online alignment tool T-Coffee (Di Tommaso et al. 2011), and pictures were created by using the Boxshade tool (Hofmann and Baron 1996) available at https://junli.netlify.app/ apps/boxshade/.

#### Bulked segregant analysis

From F2 plants that were homozygous for the *S. pennellii* allele of *Tm-1*, a selection was made for two different pools: The resistant pool (R-pool) contained 15 plants with DSI:0 and negative ToBRFV test (-), while the susceptible pool (S-pool) contained 32 plants with DSI ≥ 1 and positive ToBRFV test (+). DNA was isolated from leaf samples using the DNeasy Plant Mini kit (QIAGEN) and subsequently pooled equimolarly. Whole genome sequencing of the two pools was performed by BGI Tech Solutions (HongKong, China) using DNBSeq PE150 at 50 × coverage. Reads were mapped to the tomato Heinz SL4.0 reference genome and SNPs were identified. Subsequently, a QTL-seq analysis was performed according to Sugihara et al. (2022).

#### Genome-wide marker screening

To test linkage of the recessive resistance trait in the R-pool from the BSA, all individual plants from both pools were screened with chromosome arm-specific Indel markers discriminating between *S. lycopersicum* and *S. pennellii*, as described in Table IV from Toal et al. (2016).

## Results

### Responses of *S. pennellii* accessions to ToBRFV inoculation

**Fig. 2:**
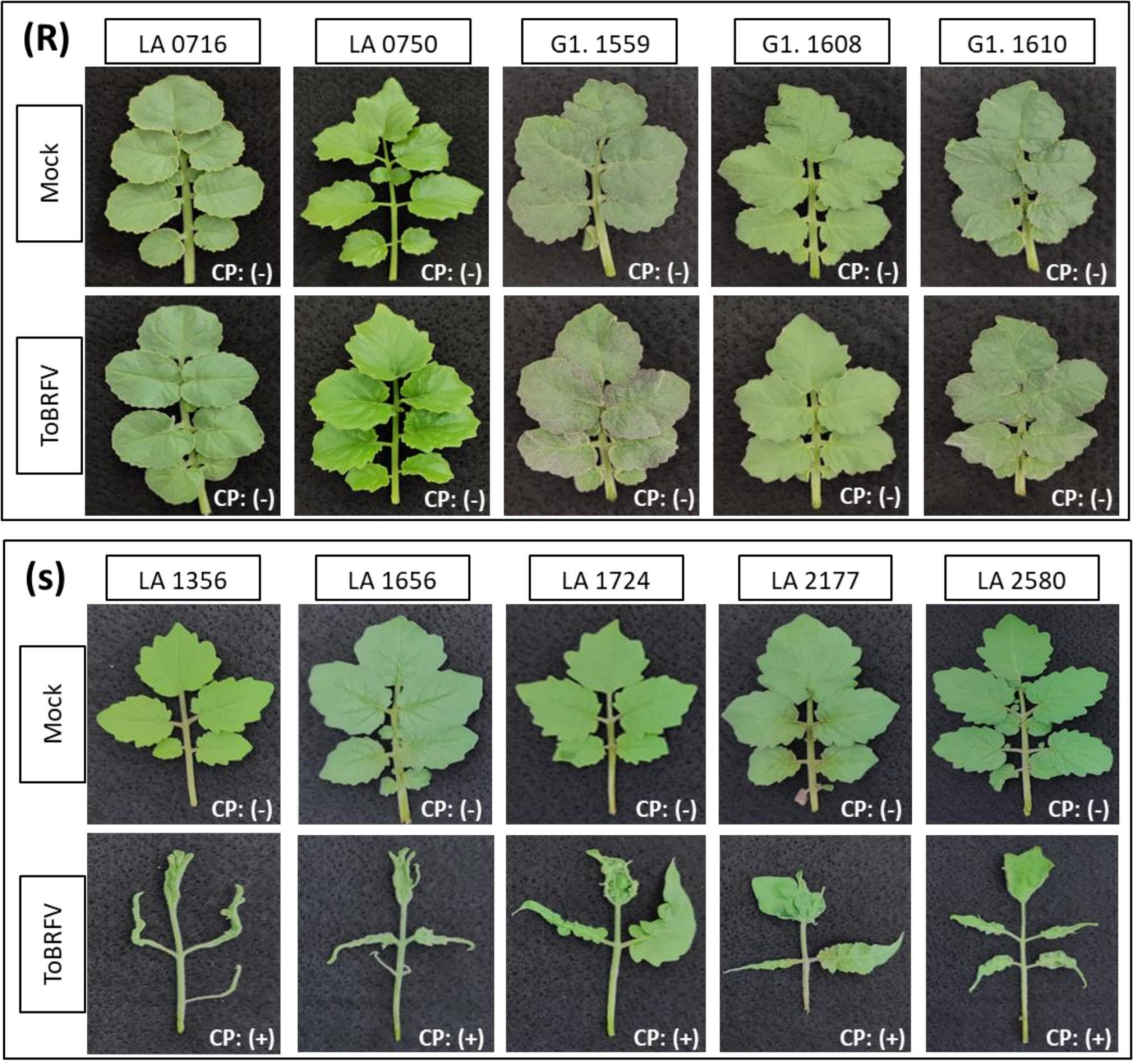
Response of *S. pennellii* accessions three weeks post-ToBRFV inoculation. Upper panel (R): Resistant accessions remained symptomless. Lower panel (S): Susceptible accessions developed severe symptoms. The top row in each panel shows leaves from mock-treated plants and the bottom row leaves from ToBRFV-inoculated plants. CP (+): Coat protein detected by ToBRFV immunostick test; CP (-): Coat protein not detected

Wild *Solanum* accessions were screened for ToBRFV resistance, resulting in the identification of multiple *S. pennellii* accessions highly resistant to ToBRFV, as well as susceptible *S. pennellii* accessions (Fig. 2). For each *S. pennellii* accession and the tomato cv. MM, ten plants were challenged with ToBRFV, while five underwent mock treatment. Three weeks post-inoculation, all ToBRFV-inoculated plants of cv. MM and five susceptible *S. pennellii* accessions (LA 1356, LA 1656, LA 1724, LA 2177, and LA 2580) displayed severe viral symptoms, including leaf shape abnormalities and stunted growth, indicating high ToBRFV inoculation efficiency. Additionally, ToBRFV immunostick testing confirmed the presence of viral coat protein (CP), indicating viral accumulation. In contrast, none of the inoculated plants from five resistant *S. pennellii* accessions (LA 0716, LA 0750, G1.1559, G1.1608, and G1.1610) exhibited symptoms, and ToBRFV immunostick testing did not detect the CP, implying low or non-existent viral accumulation. As expected, all mock-treated plants remained symptomless, with negative ToBRFV immunostick test results. The ToBRFV inoculation was repeated using new seedlings of the aforementioned accessions, yielding results consistent with the first inoculation.

### Inheritance of ToBRFV resistance derived from LA 0716 and LA 0750

**Fig. 3:**
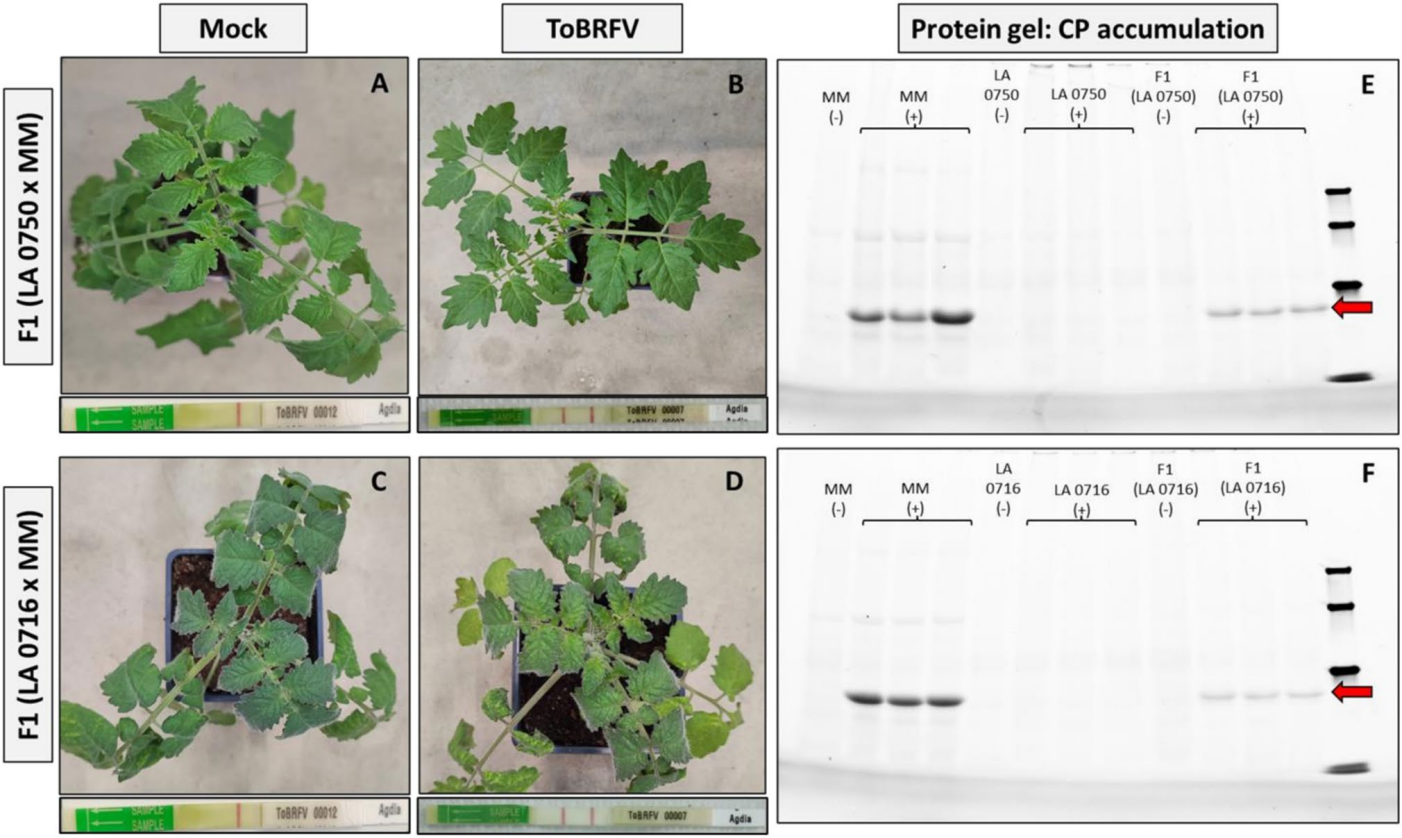
Response of F1 (LA 0716 × MM) and F1 (LA 0750 × MM) individuals four weeks post-ToBRFV inoculation and protein gel showing ToBRFV coat protein (CP) accumulation. **A** Mock-treated and **B** ToBRFV-inoculated F1 (LA 0750 × MM) plants. **C** Mock-treated and **D** ToBRFV-inoculated F1 (LA 0716 × MM) plants. Below each plant, the ToBRFV immunostick result is shown, with two red lines indicating presence of coat protein (CP) and one line indicating no detectable CP. **E** Protein gel with samples from F1 (LA 0750 × MM) plants and their parental lines. **F** Protein gel with samples from F1 (LA 0716 × MM) plants and their parental lines. (+): ToBRFV-inoculated plants, (-): mock-treated plants. The size of the ToBRFV CP protein is depicted with red arrow

To study the inheritance of ToBRFV resistance, F1 and F2 plants derived from the crosses of cv. MM with LA 0716 and LA 0750 were tested. From each F1, five plants were inoculated with ToBRFV, while another five were mock-treated and retained for F2 generation production. Three weeks post-inoculation, all mock plants remained symptomless and tested negative with the ToBRFV immunostick test, as expected. The ToBRFV-challenged F1 individuals from both crosses exhibited none to mild viral disease symptoms, such as mild mosaic and wrinkling of leaves when compared to mock plants (Fig. 3A-D). Notably, the symptoms observed in the F1 plants were less severe than those in their susceptible parent MM. Additionally, in contrast to their resistant parents (LA 0750 and LA 0716 individuals), these plants tested positive for presence of CP with the ToBRFV immunostick.

To estimate the CP accumulation of ToBRFV, protein samples extracted from F1 plants and their parental lines, inoculated with either ToBRFV or mock treatments, were visualized using protein gel electrophoresis. As expected, none of the mock-treated plants CP was detectable. CP was also not detected in the ToBRFV-challenged individuals from the LA 0716 and LA 0750 accessions. Both MM and F1 plants challenged with ToBRFV exhibited CP accumulation. However, CP accumulation in MM plants was higher than in the individuals of both F1 populations (Fig. 3E,F). These results align with the ToBRFV symptoms observed in F1 plants compared to their parental lines. Both symptoms and CP accumulation of F1 plants were at an intermediate level compared to the resistant and susceptible parents, indicating that the resistance identified in these *S. pennellii* accessions is not controlled by a single typical dominant or recessive gene.

**Fig. 4:**
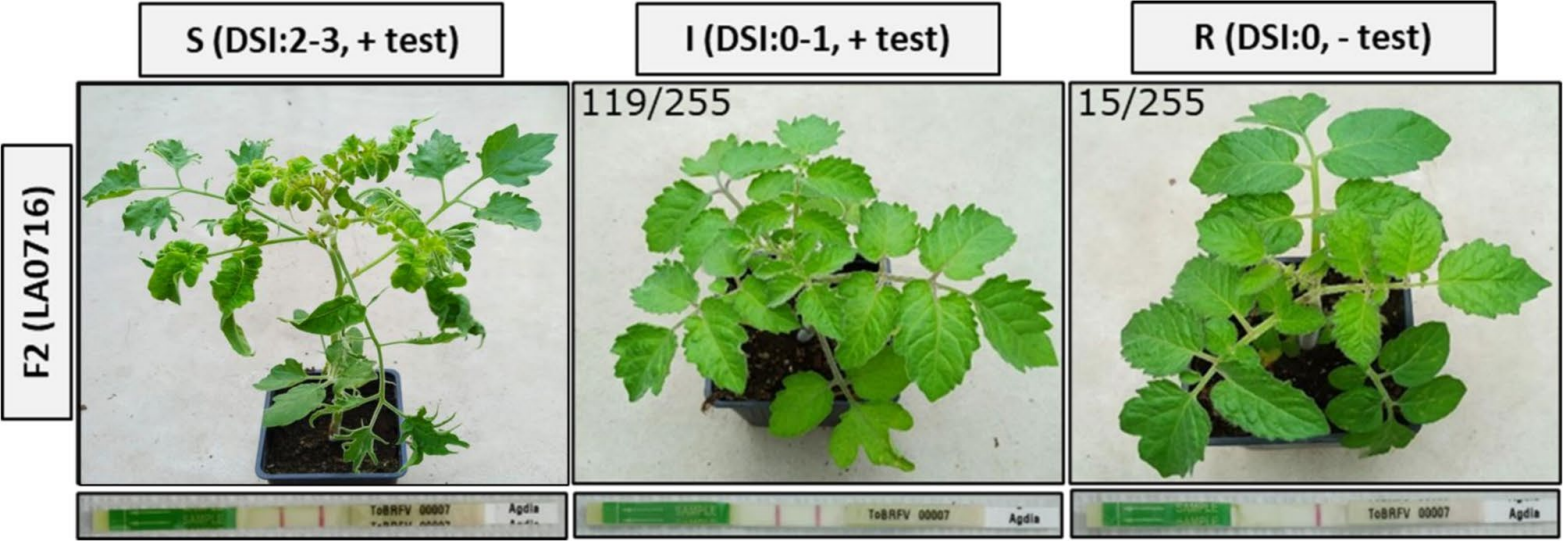
Phenotypes observed in the F2 (LA 0716) four weeks post-ToBRFV inoculation. One representative F2 plant of each phenotype [Susceptible (S), Intermediate (I) and Resistant (R)] after ToBRFV inoculation evaluated with disease severity index (DSI). At the top left of each picture, the ratio of the number of plants in the phenotypic group/total number of tested F2 (LA 0716) plants is depicted. Below each plant, the ToBRFV immunostick test result is shown, with two red lines indicating presence of ToBRFV coat protein, while one red line indicates absence of coat protein

Subsequently, 255 F2 (LA 0716) individuals were challenged with ToBRFV. Three weeks post-inoculation, the plants were phenotypically assessed using the disease severity index (DSI) outlined in Fig. 1, and the asymptomatic plants were tested for presence of CP with a ToBRFV immunostick test. The F2 (LA 0716) individuals can be categorized in three distinct phenotypic groups: susceptible (S) plants displaying severe symptoms (DSI: 2,3) and detectable CP; intermediate (I) phenotype plants showing either no or mild symptoms (DSI: 0,1) and detectable CP; and resistant (R) plants exhibiting no symptoms (DSI: 0) and no detectable CP (Fig. 4). The phenotypic segregation ratio in the F2 (LA 0716) population was 15 resistant (R): 119 intermediate (I): 121 susceptible (S). This ratio does not align with the expected 3 (R):1 (S) or 1 (R):3 (S) segregation ratios, which would be anticipated if the resistance were controlled by a single dominant or recessive gene, respectively. Therefore, the deviation from the expected segregation ratios observed in the F2 (LA 0716) supports the results obtained from the F1 plants regarding the genetic inheritance of the resistance trait. Both sets of results indicate that the resistance trait is not controlled by a single typical dominant or recessive gene.

Similar to F2 (LA 0716), 100 individuals from the F2 (LA 0750) were inoculated with ToBRFV and phenotypically assessed (Figure S1). The results were similar to those obtained with the F2 (LA 0716) population, showing a phenotypic segregation ratio in the F2 (LA 0750) population of 6 resistant (R): 47 intermediate (I): 47 susceptible (S).

### Association analysis between genetic markers for *Tm-1*, *Tm-2,* and QTL11 and ToBRFV resistance trait

**Fig. 5:**
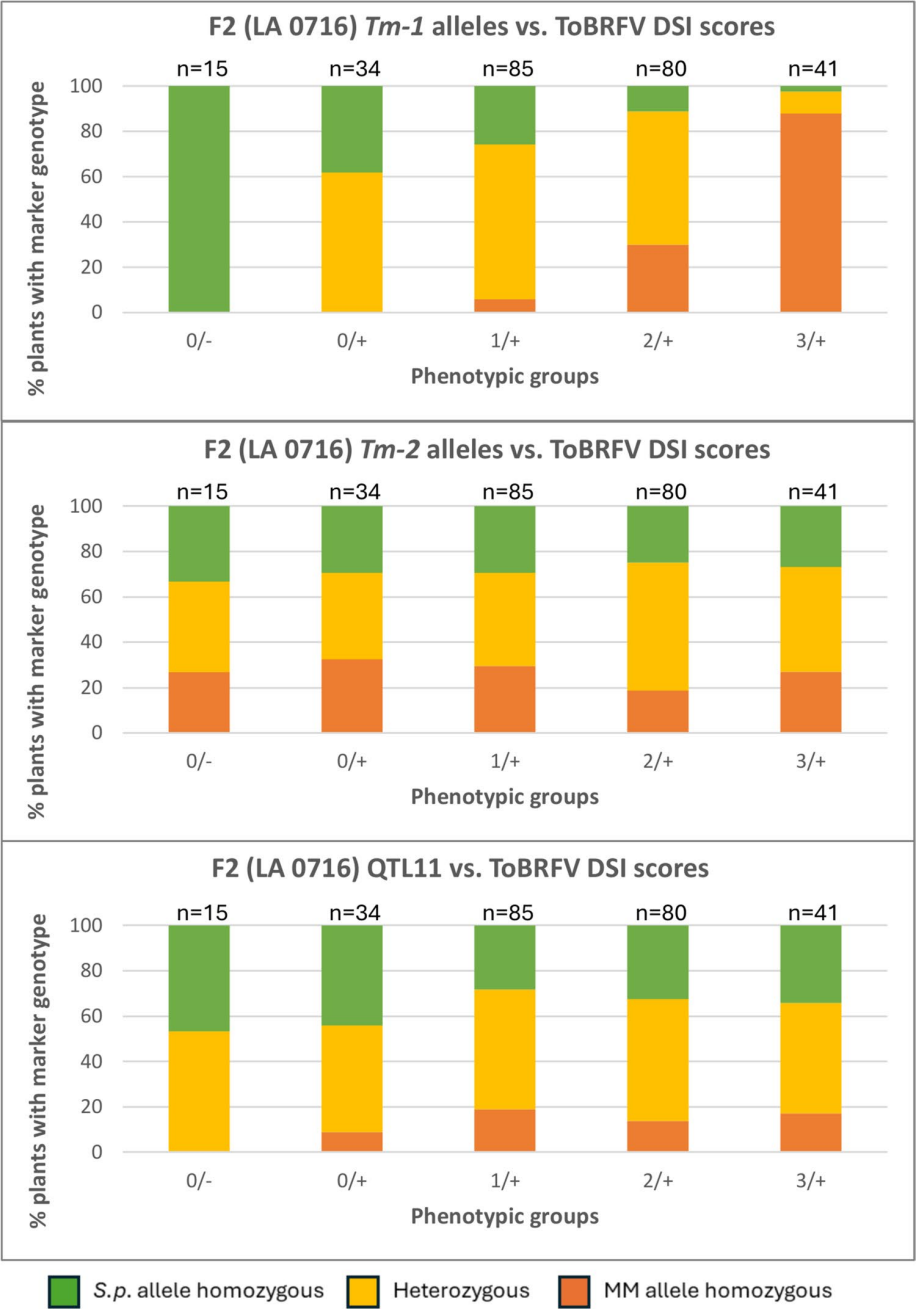
Distribution of 255 F2 (LA 0716) individuals with CAPS markers in homozygous state for the MM or the *S.p.* allele or heterozygous in five different phenotypic groups, DSI 0/no CP (0/-); DSI 0/detected CP (0/+); DSI 1/detected CP (1/+); DSI 2/detected CP (2/+); and DSI 3/detected CP (3/+). **A** Distribution of F2 individuals with *Tm-1* alleles in the different phenotypic groups. **B** Distribution of *Tm-2* alleles in the different phenotypic groups. **C** Distribution of QTL11 marker in the different phenotypic groups

Previous studies have reported three crucial loci associated with resistance to ToBRFV and other tobamoviruses: the *Tm-1* and *Tm-2* genes, a locus on chromosome 11 (QTL11), and a locus on chromosome 9 (QTL9) (Ashkenazi et al. 2020; Lindbo 2022; Zinger et al. 2021). To test whether the resistance found in *S. pennellii* LA 0716 is associated with any of these loci, cleaved amplified polymorphic sequence (CAPS) markers were developed distinguishing between MM and *S. pennellii* alleles (Table 1). Two in-gene markers were developed, one for the *Tm-1* gene (*Tm-1* marker) and a second for the *Tm-2* gene (*Tm-2* marker), and two more for the locus on chromosome 11 (QTL11 marker) and the locus on chromosome 9 (QTL9 marker). Based on polymorphisms between *S. lycopersicum* and *S. pennellii* amplicons of each marker, appropriate restriction enzymes were chosen to distinguish MM and *S. pennellii* alleles (Figure S2). The *Tm-1*, *Tm-2,* and QTL11 markers were used for the genetic analysis of the 255 F2 (LA 0716) individuals (Fig. 5). Additionally, the same markers were tested in 100 F2 (LA 0750) individuals, yielding similar results (Figure S3).

All 15 F2 (LA 0716) individuals of the resistant group (DSI:0, no detectable CP) contained the *Tm-1* allele from the *S. pennellii* resistant parent in a homozygous state (*Tm-1 S.p.*) (Fig. 5)*.* Additionally, the majority of plants exhibiting the most susceptible phenotype (DSI:3, detectable CP) were homozygous for the *Tm-1* allele from the susceptible parent (*Tm-1 MM*), suggesting the involvement of the *Tm-1* locus from *S. pennellii* in conferring resistance. Notably, we found that the frequency of plants carrying the *Tm-1 S.p*. allele decreased as the severity of symptoms increased among the phenotypic groups. Furthermore, we observed that the majority of plants with intermediate phenotypes (DSI: 0,1,2, detectable CP) carried the *Tm-1* allele in a heterozygous state. This could be explained by assuming a semidominant nature of the *Tm-1* allele from *S. pennellii*. Overall, the *Tm-1* marker is significantly related with the resistance trait, χ^2^ *(Tm-1)* [df = 8, N = 255] = 169.9, *p* = 0.00.

However, plants carrying *Tm-1 S.p.* in homozygous state are present not only in the resistant phenotypic group but also across other phenotypic groups in different frequencies. Thus, our findings suggest that fully ToBRFV-resistant plants require the presence of the *Tm-1 S.p*. locus in combination with an additional gene. Considering only the 60 plants with *Tm-1 S.p.* in homozygous state and categorizing them in plants with detectable CP (S) or non-detectable CP (R), the segregation ration is 45S:15R plants, which fits perfectly to a 3:1 segregation. This result is a strong indication that the additional gene or locus required in combination with *Tm-1 S.p.* for full ToBRFV resistance is inherited recessively.

To investigate whether full ToBRFV resistance (DSI: 0, no detectable CP) required the presence of *Tm-2*, QTL9, or QTL11 in addition to *Tm-1*, we analyzed markers for these loci in a subset of 60 F₂ (LA 0716) individuals that were homozygous for the *S.p. Tm-1* allele (Figure S4). None of the three markers showed a significant association with the resistance phenotype (χ^2^ (*Tm-2*) [df = 2, N = 60] = 0.9, *p* = 0.63; χ^2^ (QTL9) [df = 2, N = 60] = 1.46, *p* = 0.48; χ^2^ (QTL11) [df = 2, N = 60] = 1.23, *p* = 0.54). Regardless of whether these individuals were homozygous for the *S.p.* or MM alleles, or heterozygous at these loci, they were distributed across the two phenotypic groups without significant differences. Thus, no evidence of association of any of these loci with ToBRFV resistance or susceptibility was observed.

Interestingly, the analysis of QTL9 and *Tm-2*, both located on chromosome 9 but on opposite arms, revealed high co-segregation, with 55 out of 60 plants sharing identical genotypes at both loci. This strong linkage suggests that chromosome 9 may contain a recombination cold spot in this population. As a result, a substantial portion of chromosome 9, including the region flanked by *Tm-2* and QTL9, can likely be excluded as the location of the second gene required for full ToBRFV resistance in *S. pennellii* accession LA 0716.

In an attempt to identify the second locus required for full ToBRFV resistance from *S. pennellii* LA 0716, a bulked segregant analysis (BSA) was performed. For this, a QTL-seq analysis (Sugihara et al. 2022) was carried out on SNPs identified after whole genome sequencing of two DNA pools of F2 plants homozygous for the *S.p.* allele of *Tm-1*, but contrasting in resistance. The R-pool contained the 15 plants showing no symptoms and no ToBRFV CP (no viral accumulation), while the S-pool contained 32 plants showing symptoms (DSI ≥ 1) and presence of ToBRFV CP. However, the QTL-seq results did not show any significant QTL on any of the chromosomes (Figure S5).

Additionally, we screened the individual plants of the BSA pools (in total 47 plants) with 18 Indel markers distinguishing between *S. lycopersicum* and *S. pennellii* LA 0716 alleles as described by Toal et al. (2016), for all chromosome arms that had not been tested yet. None of these markers displayed a correlation with full resistance (Table S1).

### VIGS-mediated functional analysis of *Tm* genes in *S. pennellii* accessions in relation to ToBRFV resistance

To establish that *Tm-1*, and not another gene linked to the *Tm-1* locus, is involved in resistance to ToBRFV from *S. pennellii*, we employed VIGS to silence *Tm-1* in plants from all five identified resistant *S. pennellii* accessions. Three distinct genes were silenced: *Tm-1*, suspected as the candidate gene associated with resistance, while *Tm-2* served as a negative control and the *E. coli GUS* gene, with no homolog in *Solanum* species, functioned as a second negative control. From each resistant *S. pennellii* accession five plants at the cotyledon stage were subjected to agro-inoculation with the VIGS constructs. Two weeks post-VIGS inoculation, the plants exhibiting silenced genes were challenged with ToBRFV. Additionally, non-VIGS-treated plants from the same accessions and at the same developmental stage as the VIGS-treated plants were inoculated with ToBRFV as controls. Finally, some plants from each *S. pennellii* accession were kept as mock, without VIGS treatment and no ToBRFV inoculation, to serve as additional controls.

**Fig. 6:**
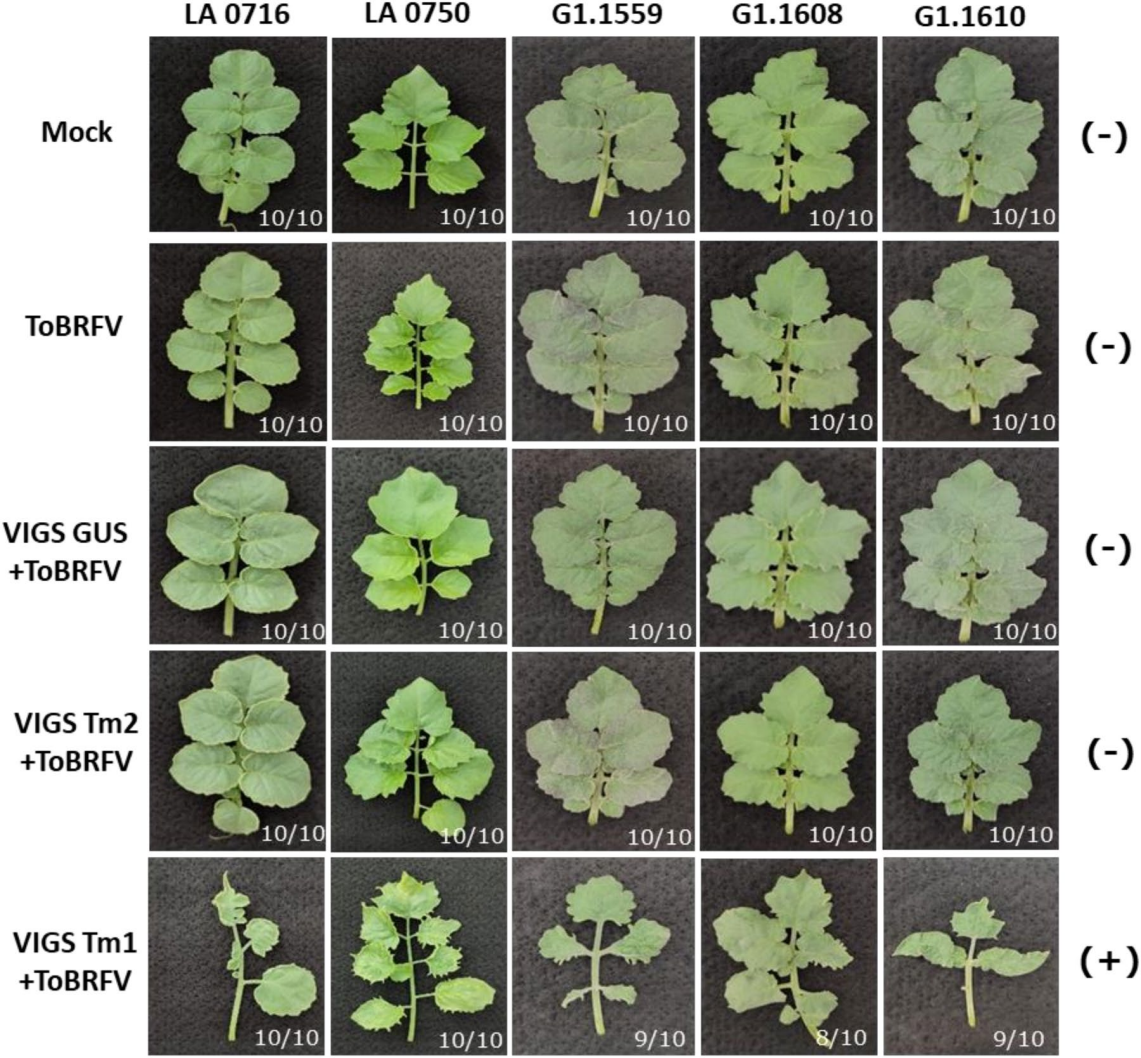
VIGS-mediated functional analysis of *Tm* genes in individuals of five ToBRFV-resistant *S. pennellii* accessions regarding ToBRFV symptom development. Each column represents plants from different *S. pennellii* accessions. The top row shows leaves from plants with no VIGS and no ToBRFV inoculation (Mock). The second row depicts leaves from plants inoculated with ToBRFV but not subjected to VIGS. The third row presents leaves from GUS-silenced plants inoculated with ToBRFV. The fourth row displays leaves from *Tm-2* silenced plants inoculated with ToBRFV. The last row shows leaves from *Tm-1* silenced plants, also inoculated with ToBRFV. To the right of each column, results of the ToBRFV CP test from all the plants of the row are indicated, where (+) denotes detected CP and (−) denotes no detected CP. At the bottom right of each image, the ratio of plants showing the specific phenotype to the total number of *S. pennellii* accession plants with the corresponding treatment is provided

Three weeks post-ToBRFV inoculation, plants from control treatments, *Tm-2* and *GUS* silencing, showed no symptoms and exhibited normal growth, similar to non-VIGS-treated plants inoculated with ToBRFV, as well as mock-treated plants (without VIGS or ToBRFV inoculation) (Fig. 6). Hence, the TRV VIGS system by itself seems not to influence ToBRFV resistance, and thus to be an appropriate tool for functional analysis of ToBRFV resistance genes. We observed that all *Tm-1-*silenced plants from the resistant *S. pennellii* accessions displayed severe ToBRFV symptoms. Furthermore, all the plants from the various treatments were tested with ToBRFV immunostick for presence of CP. The CP was only detected in plants in which *Tm-1* was silenced, while it was not detected in any of the other plants. Both, the presence of symptoms and the detection of CP in the *Tm-1* silenced plants, confirm the involvement of the *Tm-1* gene in conferring resistance to ToBRFV in the resistant *S. pennellii* accessions.

### *Tm-1 *allelic variation among *Solanum* accessions

**Fig. 7:**
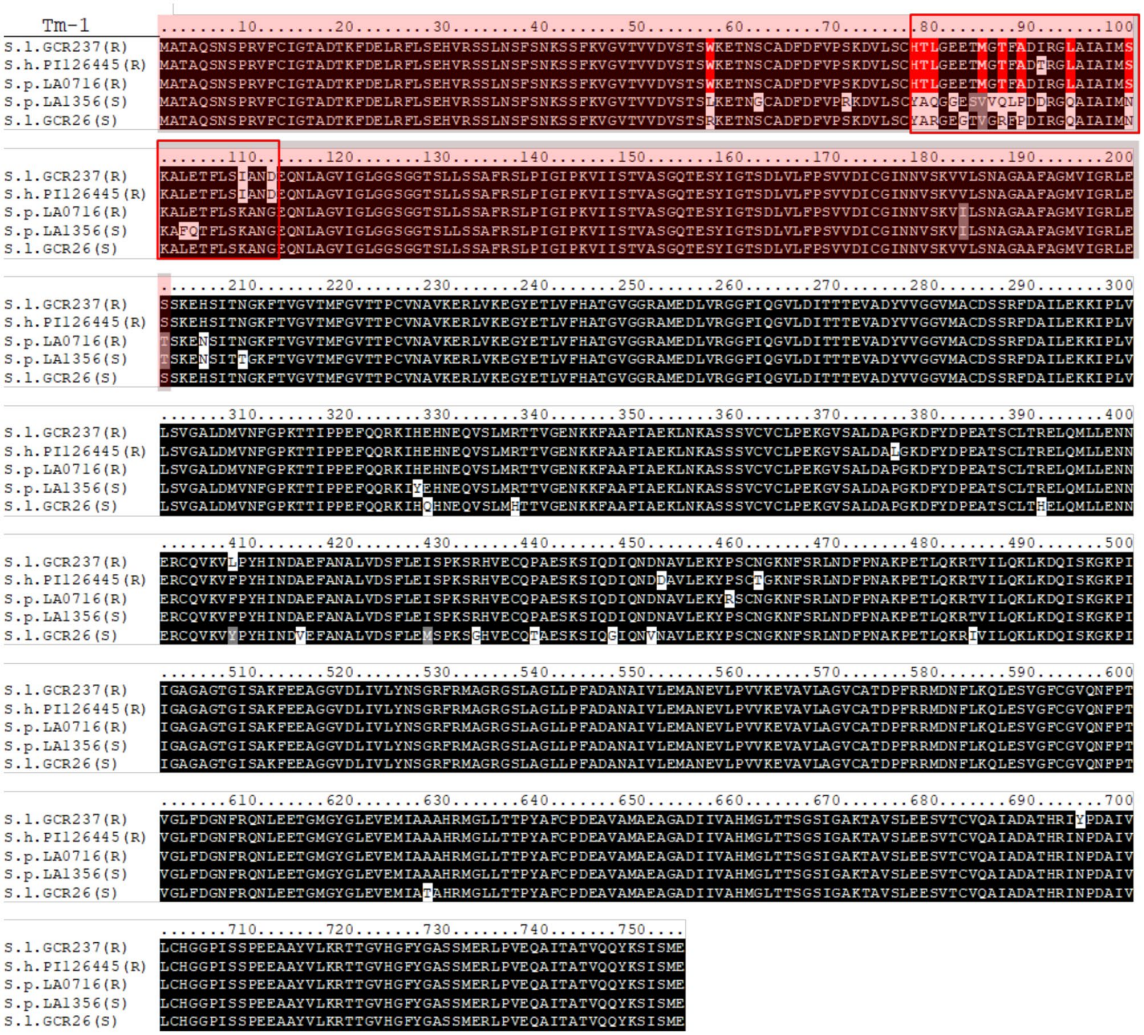
Alignment of the Tm-1 protein sequence from resistant *S. pennellii* LA 0716 [S.p. LA 0716 (R)], the original *Tm-1* donor *S. habrochaites* PI126445 [S.h. PI126445 (R)], the resistant *S. lycopersicum* GCR237 [S.l. GCR237 (R)], the susceptible *S. pennellii* LA1356 [S.p. LA1356 (S)] and the susceptible *S. lycopersicum* GCR26 [S.l. GCR26 (S)]. The red-shaded region represents the 201-aa peptide that binds ToMV replicase and inhibits viral replication in vitro. The boxed region highlights the region under positive selection for ToMV resistance in *S. habrochaites* accessions. Amino acids associated with the ToBRFV resistance, shared among resistant Tm-1 proteins and differ from susceptible variants, are highlighted in red

*Tm-1* alleles were amplified by PCR using cDNA from all the tested *S. pennellii* accessions. Sequences were aligned together with the *Tm-1* (*Sopen02g013570*) sequence annotated in the *S. pennellii* (LA 0716) genome retrieved by Sol genomics database (Bolger et al. 2014). The *Tm-1* alleles of all resistant *S. pennellii* accessions are 100% identical with the annotated *Tm-1* (*Sopen02g013570*) gene; therefore, only one *Tm-1* sequence from the resistant *S. pennellii* accessions was used to predict its protein sequence. All susceptible *S. pennellii* accessions carry an identical *tm-1* allele which differs from the *Tm-1* allele found in the resistant *S. pennellii* accessions. Moreover, the amino acid (aa) sequences of ToMV resistance allele *Tm-1* (GenBank: BAF75724; S.l. GCR237), the ToMV-susceptible allele *tm-1* (GenBank: BAF75725; S.l. GCR26) from *S. lycopersicum* and the Tm-1 from the *S. habrochaites* accession PI126445 (NCBI: AB713135) were retrieved from the NCBI database. The accession PI126445 is the donor of the original ToMV resistant *Tm-1* allele (Ishibashi et al. 2012). The aa sequences of all the above *Tm-1* alleles were aligned, and sequence differences were detected (Fig. 7).

Ishibashi et al. (2014) demonstrated that the first 201 amino acids (aa) of Tm-1 can bind the ToMV replication (REP) protein and inhibit replication in vitro (Fig. 7, red-shaded region). Additionally, Ishibashi et al. (2012) identified the region spanning aa 78–112 as being under positive selection in *S. habrochaites* Tm-1 in response to resistance-breaking ToMV strains, highlighting its functional importance (Fig. 7, boxed region). Notably, nine amino acids distinguish ToBRFV-resistant Tm-1 proteins from susceptible tm-1 variants, all within the first 201 aa, with eight located in the positively selected region for ToMV (aa 78–112) at positions 57, 78, 79, 80, 85, 87, 89, 94, and 100 (Fig. 7, red-highlighted). Additionally, a unique amino acid at position 459 in the Tm-1 protein of ToBRFV-resistant *S. pennellii* distinguishes it from all other Tm-1 variants.

## Discussion

### ToBRFV: A global threat and its control challenges

Tomato brown rugose fruit virus (ToBRFV) has rapidly become a significant concern for tomato production worldwide due to its devastating impact on crop yields (Caruso et al. 2022). The virus has been responsible for substantial economic losses across major tomato-growing regions, jeopardizing both large-scale agricultural operations and smallholder farms. Its rapid spread has exacerbated these challenges, making ToBRFV a global agricultural threat (Zhang et al. 2022). Compounding this issue, commonly used resistance genes in tomato which confer resistance to tobamoviruses, such as *Tm-2/Tm-2*^*2*^, have been proven ineffective against ToBRFV (Hak and Spiegelman 2021; Jaiswal et al. 2024; Luria et al. 2018; Zinger et al. 2021). As a result, the search for new resistance traits within the tomato germplasm has become a critical focus for researchers and breeders alike, aiming to mitigate the devastating effects of this pathogen on tomato production. These efforts have led to the identification and use of some ToBRFV resistance and tolerance traits in *Solanum* species. However, ToBRFV isolates that can overcome some of these resistance traits have already been reported. For instance, a single amino acid mutation in the movement protein (MP) of the ToBRFV_G78_RB isolate (NCBI: MZ438228.1) has been shown to break the resistance of a commercial tomato cultivar (Zisi et al. 2024), which is conferred by a dominant NBS-LRR gene located on chromosome 8 derived from *S. habrochaites* accession LYC4943 (Ykema et al. 2020). These findings illustrate the rapid resistance-breaking potential of RNA viruses like ToBRFV, as also described by Rubio et al. (2020), and emphasize the urgent need for continued efforts to discover additional resistance traits that can provide durable protection against this viral threat.

### *Tm-2 *is not involved in the *S. pennellii* resistance

Several studies have shown that both alleles, *Tm-2* and *Tm-2*^*2*^, originally derived from *S. peruvianum* accessions and known for conferring resistance to ToMV, are ineffective against ToBRFV (Hak and Spiegelman 2021; Yan et al 2021). However, Lindbo (2022) demonstrated that specific engineered amino acid substitutions can restore *Tm-2*^*2*^ functionality against ToBRFV. Specifically, amino acid substitutions at position 822 from asparagine (S) to cysteine (C), phenylalanine (F), methionine (M), tyrosine (Y), or tryptophan (W); at position 825 from serine (G) to histidine (H), lysine (K), or threonine (T); and at position 848 from cysteine (F) to arginine (R). While the *S. pennellii* (LA 0716) Tm-2^2^ orthologue (Sopen09g035210) protein shows significant differences from the traditional Tm-2^2^ (NCBI: AAQ10736) (ID%: 74.15), these amino acid variations do not coincide with the substitutions conferring ToBRFV resistance (Figure S6). In our study, CAPS marker analysis of F2 (LA 0716) individuals confirmed that the *Tm-2* locus is not linked to the resistance trait. Furthermore, silencing *Tm-2* orthologues in all tested *S. pennellii* accessions did not alter their response to ToBRFV (Fig. 6), reinforcing the conclusion that the *Tm-2* alleles in *S. pennellii* do not play a role in ToBRFV resistance.

### The role of *Tm-1* in *S. pennellii* resistance

Although the original ToMV resistance *Tm-1* allele from *S. habrochaites* was initially reported as ineffective against ToBRFV, later studies identified its involvement in ToBRFV resistance (Hak and Spiegelman 2021; Jaiswal et al. 2024; Luria et al. 2018; Zinger et al. 2021, 2025). The present study revealed that all resistant F2 (LA 0716) and F2 (LA 0750) plants possessed the *S. pennellii Tm-1* allele in a homozygous state, as confirmed by CAPS marker analysis, suggesting that for the resistant *S. pennellii* accessions LA 0716 and LA 0750, the *Tm-1* locus plays a crucial role in ToBRFV resistance. VIGS assays further confirmed the involvement of the *Tm-1* gene in ToBRFV resistance across all tested resistant *S. pennellii* accessions, as silencing *Tm-1* rendered these plants susceptible to ToBRFV infection.

Ishibashi (2010) demonstrated that allelic variants of *Tm-1* can interact with different tobamoviruses and inhibit their proliferation; for instance, the ToMV-susceptible allele of *Tm-1* (*S.l. tm-1*) functions as an inhibitor of RNA replication for other tobamoviruses, such as tobacco mild green mosaic virus (TMGMV) and Pepper mild mottle virus (PMMoV). Based on our findings that *S. pennellii Tm-1 (S.p. Tm-1)* plays a major role in ToBRFV resistance and considering the broader interactions of *Tm-1* alleles with various tobamoviruses, we speculate that the *S.p. Tm-1* is an allelic variant of this gene capable of interacting with the ToBRFV REP protein, thereby halting its proliferation.

After comparing the aa sequence of Tm-1 from the susceptible and resistant *S. pennellii* accessions identified in this study, along with other ToBRFV-resistant and -susceptible Tm-1 variants from the literature, we identified nine unique aa associated with resistance. Additionally, a unique aa specific to the ToBRFV-resistant *S. pennellii* accessions was identified at position 459. Notably, all nine ToBRFV resistance-associated aa are located within the first 201 aa, a region previously shown to be sufficient for inhibiting ToMV replication in vitro (Ishibashi et al. 2014). Eight of these nine aa reside within the *Tm-1* region spanning aa 78–112, which was identified as a positively selected site against ToMV (Ishibashi et al. 2012). The exclusive presence of these aa in resistance-conferring Tm-1 variants and their location within functionally critical regions strongly suggest their key role in ToBRFV resistance. The unique aa at position 459 in the Tm-1 protein of ToBRFV-resistant *S. pennellii* is not located in a region previously described as important for its function against tobamoviruses. However, this does not rule out its potential contribution to improved affinity in the interaction between Tm-1 and ToBRFV REP.

### An additional locus from *S. pennellii* is required for full ToBRFV resistance

Interestingly, some plants from the F2 (LA 0716) population displayed symptoms and/or CP accumulation despite having the *S.p. Tm-1* allele in a homozygous state. This finding suggests that *Tm-1* alone is not sufficient to confer complete resistance, indicating the involvement of an additional locus. The segregation ratio of only those F2 (LA 0716) individuals with the homozygous *S.p. Tm-1* allele followed a 3 susceptible (S): 1 resistant (R) pattern, which is characteristic of traits inherited in a recessive manner. Also, only 15 out of 255 F2 (LA 0716) and 6 out of 100 F2 (LA 0750) plants show full resistance, which fits a 1:15 ratio (χ^2^ = 0.059 [*p* = 0.81] and χ^2^ = 0.011 [*p* = 0.92], respectively) expected for two unlinked loci of which the resistant alleles both need to be present in homozygous state. This observation implies that the additional locus, which works in conjunction with *Tm-1* to confer ToBRFV resistance, is likely inherited recessively.

Previous studies have also suggested the involvement of *Tm-1* gene in combination with additional loci in ToBRFV resistance. Ashkenazi et al. (2020) reported that the ToBRFV resistance trait in a *S. lycopersicum* cultivar was due to the *Tm-1* gene combined with a recessively inherited locus on chromosome 11 and/or chromosome 9. Similarly, Zinger et al. (2021) described a resistance trait in *S. lycopersicum*, conferred by the combination of *Tm-1* locus with a recessive locus on chromosome 11. Another study pointed the recessive gene *SICCA1* (*Solyc11g018770*), located within the chromosome 11 loci mentioned in the other two studies, as the additional resistance contributor alongside *Tm-1* (Kalisvaart et al. 2021).

To explore this further, we used a CAPS marker located within the chromosome 11 loci described by Zinger et al. (2021) and Ashkenazi et al. (2020) in the F2 (LA 0716) individuals. Our results showed that this marker is not linked with the resistance trait found in the *S. pennellii* LA 0716 accession, demonstrating that the resistance in *S. pennellii* is governed by a different additional locus. Since the *SICCA1* gene is located near the CAPS marker on chromosome 11, and our results show that this marker is not linked to the resistance in *S. pennellii*, we could conclude that *SICCA1* is not the additional resistance contributor in resistant *S. pennellii* plants.

Furthermore, the *Tm-2* gene, located on chromosome 9, lies within a region known to be a cold spot for recombination (Fuentes et al. 2024). We tested the QTL9 CAPS marker positioned at the end of the opposite arm of chromosome 9, far from the *Tm-2* region, in a subset of F2 (LA 0716) individuals. This marker showed co-segregation with the *Tm-2* marker, reinforcing the idea that chromosome 9 is a recombination cold spot. However, both markers did not show co-segregation with the resistance trait. Consequently, we can conclude that these loci on chromosome 9 are not linked to the resistance found in *S. pennellii* LA 0716.

These observations suggest that the locus in *S. pennellii* LA 0716, which works alongside *Tm-1* to confer ToBRFV resistance, does not overlap with the loci described in the two key studies that identified ToBRFV resistance involving *Tm-1*. This indicates the potential novelty of the additional gene in *S. pennellii* LA 0716, which, in combination with *S.p. Tm-1*, confers robust ToBRFV resistance. Topcu et al. (2025) identified 14 QTL for ToBRFV tolerance by GWAS involving *S. lycopersicum* var. *lycopersicum*, *S. lycopersicum* var. *cerasiforme,* and *S. pimpinellifolium* accessions. In addition to QTLs on chromosome 11, they observed QTLs on chromosomes 1, 2, 3, 6, 7, 10, and 12.

Our results do not clarify whether the additional gene works synergistically with *Tm-1* to inhibit virus proliferation or if it functions through an independent resistance or tolerance mechanism. This independent mechanism, combined with *Tm-1* inhibition of viral replication, may collectively confer complete resistance to ToBRFV. Interestingly, Ishibashi (2010) demonstrated that tomato is a non-host for the tobamovirus TMGMV due to the presence of a naturally occurring *Tm-1* allele (*S.l. tm-1*). A TMGMV strain later emerged with reduced interaction with *S.l. tm-1*, allowing it to replicate in tomato protoplasts as efficiently as ToMV. However, in tomato leaves the proliferation of this TMGMV strain was significantly lower, suggesting the presence of an additional inhibitory mechanism that prevents the virus from spreading intercellularly. In this case, *Tm-1* and the secondary mechanism appeared to act independently against TMGMV. A similar scenario could explain the ToBRFV resistance observed in *S. pennellii* accessions, where the *S.p. Tm-1* allele and the additional locus might function independently or in combination. We have not been able to identify the additional locus yet. Although the segregation ratio suggests the requirement of one recessive locus in addition to homozygosity of the *S.p. Tm-1* allele for full ToBRFV resistance, we cannot exclude the possibility of a more complex genetic model. Further studies are needed to elucidate the mechanisms behind *S.p. Tm-1* and the additional locus/loci in conferring ToBRFV resistance.

### Discrepancies in *S. pennellii* LA 0716 ToBRFV resistance in different studies

In this study, we found that five *Solanum pennellii* accessions exhibited high resistance to ToBRFV. However, previous studies have reported conflicting results regarding the resistance of *S. pennellii*, specifically the LA 0716 accession, to ToBRFV. Jewehan et al. (2022b) classified *S. pennellii* as completely susceptible to ToBRFV while Kabas et al. (2022) characterized the LA 0716 accession as ToBRFV-tolerant, observing mild symptoms. Topcu et al. (2025) mentioned that LA 0716 did not exhibit any ToBRFV symptoms, but was considered highly tolerant as ToBRFV virus could be detected by RT-qPCR. These divergences could be attributed to genetic variation within the tested *S. pennellii* accessions, differences in the ToBRFV isolates used, or varying environmental conditions. Most of the *S. pennellii* accessions, including LA 0716, are self-compatible and predominantly self-pollinated (Flores-Hernández et al. 2018), making them genetically homogeneous, with limited variation (Mercer and Perales 2010; Rick and Tanksley 1981). Thus, genetic differences within the LA 0716 accession are unlikely to explain the observed differences in ToBRFV infectivity.

Another potential factor is the use of different ToBRFV isolates. Jewehan et al. (2022b) used the ToBRFV-Tom2-Jo isolate (GenBank Accession No. MZ323110), while Kabas et al. (2022) and Topcu et al. (2025) used the ToBRFV-Ant-Tom isolate (GenBank Accession No. MT107885). In our study, we used the ToBRFV-NVWA isolate (GenBank Accession No. MN882011). The CP amino acid sequence is identical across the three ToBRFV isolates (Figure S7), indicating that CP is unlikely to contribute to the observed differences in infectivity.

A comparison of REP protein sequences showed that ToBRFV-NVWA differs from Ant-Tom and Tom2-Jo by two aa at positions 1206 and 1363, while NVWA and Ant-Tom differ from Tom2-Jo by one aa at position 984 (Figure S8). The helicase domain (aa 801–1116) is the most crucial region of REP for interaction with Tm-1 (Ishibashi 2010; Ishibashi et al. 2012; Ishibashi and Ishikawa 2014). The differences at positions 1206 and 1363 are outside this domain, whereas the difference at position 984, found in Tom2-Jo, is within the helicase domain. This aa difference in Tom2-Jo may be responsible for breaking *S. pennellii Tm-1*-mediated resistance. This hypothesis is supported by a recent study (Kubota et al. 2024), which identified several aa substitutions, including one at position 984, in the helicase domain of ToBRFV isolates capable of breaking the resistance conferred by the GCR237 *Tm-1* allele. Hence, this observation can not only explain the differences in ToBRFV response of plants from the same *S. pennellii* accession (LA 0716) among the different studies, but also suggests that the aa substitution at position 984 of ToBRFV REP alone may be sufficient to break the resistance conferred by *Tm-1* alleles.

Moreover, ToBRFV-Ant-Tom lacks the first 26 aa of the small subunit of the REP protein. While this missing segment is not part of the helicase domain, it may influence the protein’s secondary structure and potentially affect its function. Furthermore, two aa differences were observed between the movement protein (MP) of Tom2-Jo and NVWA, and Ant-Tom (Figure S9). Our findings suggest that the *S. pennellii* resistance trait involves *Tm-1* in combination with an additional locus. The two amino acid differences in the MP between these isolates might interfere with the contribution of this additional locus. That could be another explanation why Jewehan et al. (2022b) found the LA 0716 accession susceptible.

Environmental factors may also contribute to the discrepancies in ToBRFV infectivity. Jewehan et al. (2022a) demonstrated that *S. habrochaites* and *S. peruvianum* accessions, resistant to ToBRFV at 24 °C, displayed severe symptoms when inoculated at 33°C. Similarly, the resistance conferred by *Tm-1* against ToMV is temperature-dependent (Fraser and Loughlin 1982). *Tm-1* plants inhibited TMV proliferation at 25 °C but lost this ability at 33°C. In our study, plants were inoculated at 20 °C, whereas Kabas et al. (2022) conducted their inoculations and 28 °C, respectively. The higher temperature used in their study may have reduced the efficacy of *S.p. Tm-1* against ToBRFV, contributing to the differences in the plant’s response to the viral infection. It would be valuable to further investigate the impact of elevated temperatures on ToBRFV resistance in *S. pennellii* accessions, particularly those tested in our study.

## Conclusion

Tomato brown rugose fruit virus (ToBRFV) threatens global tomato production, with emerging new isolates that overcome resistance genes. This highlights the urgent need for continued research not only to identify and exploit individual resistance genes but to discover multiple genes with diverse resistance mechanisms. By combining these genes, we can develop multilayered, durable resistance to counter this viral threat more effectively. Our study identified *S. pennellii* accessions as a valuable source of ToBRFV resistance, with the *S. pennellii Tm-1* allele playing a key role. Moreover, comparison of the *Tm-1* amino acid sequences from resistant and susceptible accessions revealed nine amino acid differences that are putatively involved in the resistance function of *Tm-1* against ToBRFV. Additionally, we demonstrated that complete resistance requires a secondary locus, likely inherited recessively. Notably, this locus does not overlap with those previously reported from other studies to act alongside *Tm-1*, suggesting its potential as a novel factor in ToBRFV resistance. Our findings can be of great value for breeding programs focused on developing tomato cultivars with strong, durable resistance to ToBRFV.

## Supplementary Information

Below is the link to the electronic supplementary material.

Supplementary file1 (DOCX 2447 KB)

## Data Availability

The data generated and analyzed supporting the findings of the current work are available within the manuscript and its supplementary information files.
