# Supplementary material for: Tm-1 back in business: an allele from Solanum pennellii accessions plays a major role in ToBRFV resistance
Source: Theor Appl Genet. 2025 Sep 12;138(10):248. doi: 10.1007/s00122-025-05036-1 (PMC12432084; doi:10.1007/s00122-025-05036-1)
Supplement: Supplementary file 1 — Supplementary file1 (DOCX 2447 KB) [file 122_2025_5036_MOESM1_ESM.docx]

***Tm-1* back in business: an allele from *Solanum pennellii* accessions plays a major role in ToBRFV resistance.**

Romanos Zois, Mireille van Damme, Martin Verbeek, Luuk D.H. Veenendaal, Yuling Bai, Anne-Marie A. Wolters

**Supplementary Figures and Table**


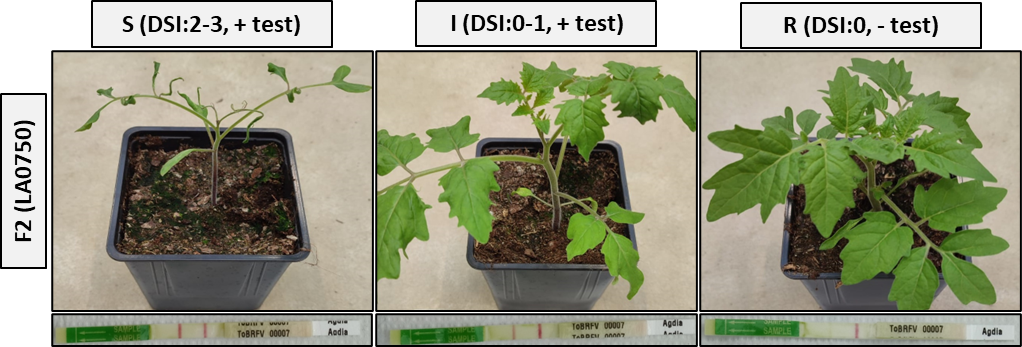


**Figure S1** Phenotypes observed in the F2 (LA 0750) four weeks post ToBRFV inoculation. One representative F2 plant of each phenotype [47 plants Susceptible (S), 47 plants Intermediate (I) and 6 plants Resistant (R)] after ToBRFV inoculation evaluated with disease severity index (DSI). Below each plant, the ToBRFV immunostick test result is shown, with two red lines indicating presence of ToBRFV coat protein while one red line indicates absence of coat protein.


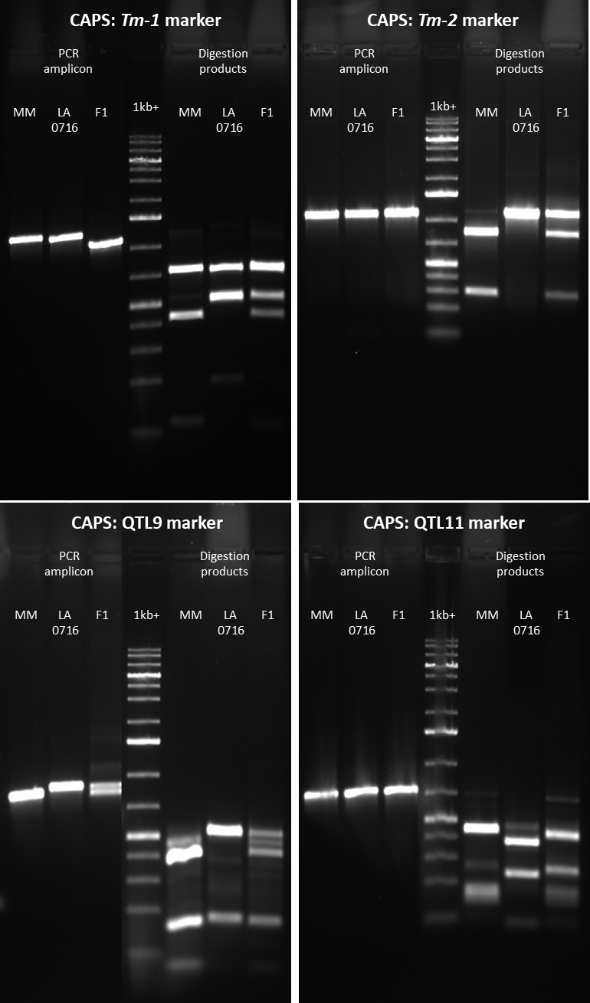


**Figure S2** Gel image of the CAPS markers. The three bands to the right of the DNA ladder (GeneRuler 1 kb Plus DNA Ladder, ThermoFisher Scientific) represent PCR products amplified using specific primers for each locus. To the left of the 1kb+ ladder are the corresponding PCR products digested with specific restriction enzymes. Primers and restriction enzymes are provided in Table 1.


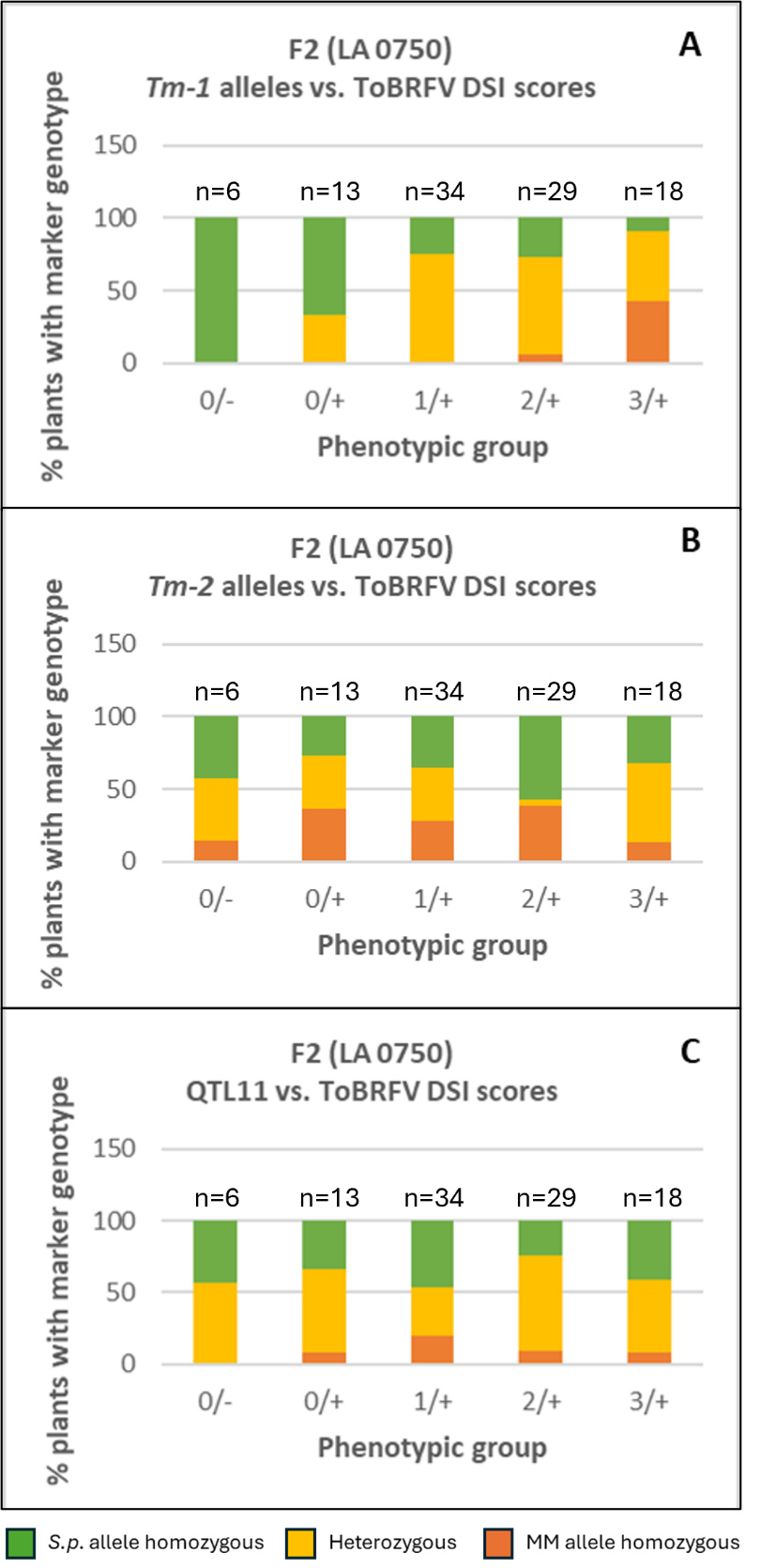


**Figure S3** Distribution of F2 (LA 0750) individuals with CAPS marker in homozygous state for the MM or the S.p. allele or heterozygous in five different phenotypic groups, DSI (Disease Severity Index) 0/no Coat Protein (CP) ( 0/-), DSI 0/ detected CP (0/+), DSI 1/ detected CP (1/+), DSI 2/ detected CP (2/+), and DSI 3/detected CP (3/+). **A.** CAPS marker Tm-1, **B.** CAPS marker Tm-2, **C.** CAPS marker QTL11.

**
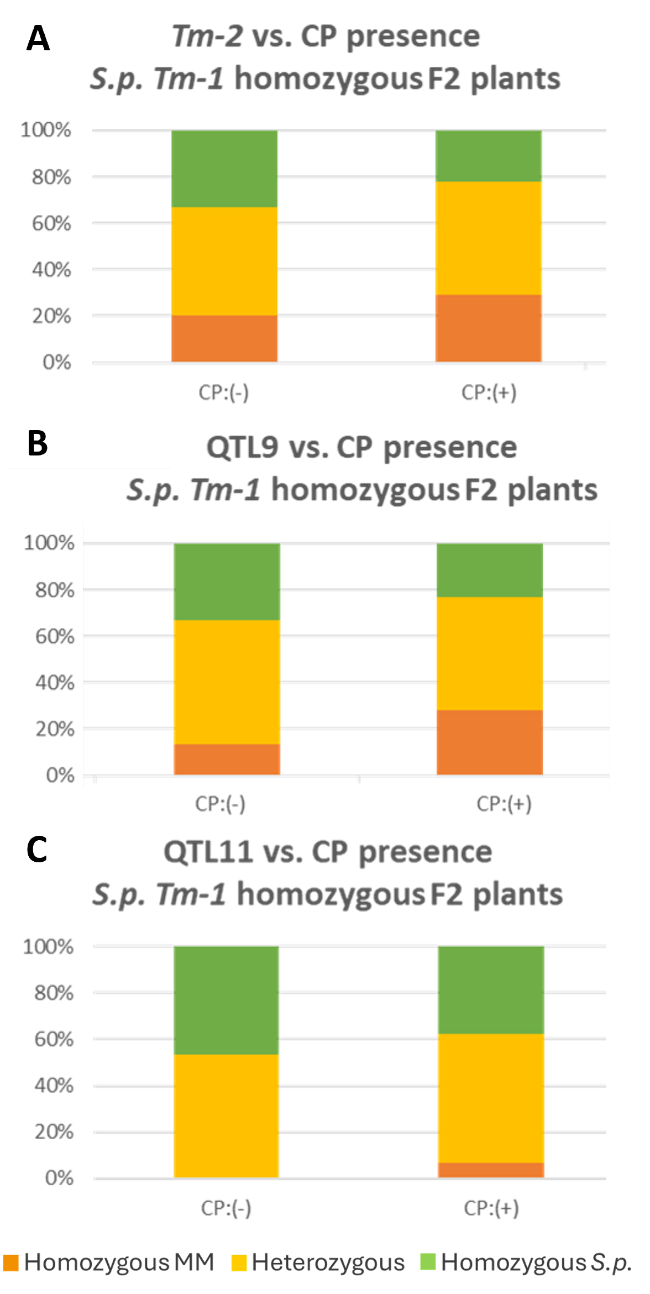
**

**Figure S4** Percentage of 60 F2 (LA 0716) individuals homozygous for the Moneymaker (MM) allele (orange), heterozygous (yellow), or homozygous for the LA 0716 *S.p.* allele (green) for three different CAPS markers. The 60 F2 plants are all homozygous for the *S.p. Tm-1* allele, but divided in two phenotypic groups; 15 resistant F2 plants with no detected ToBRFV coat protein CP:(-), and 45 F2 plants with detected CP:(+). **A.** Distribution of *Tm-2* marker. **B.** Distribution of QTL9 marker. **C.** Distribution of QTL11 marker.

**
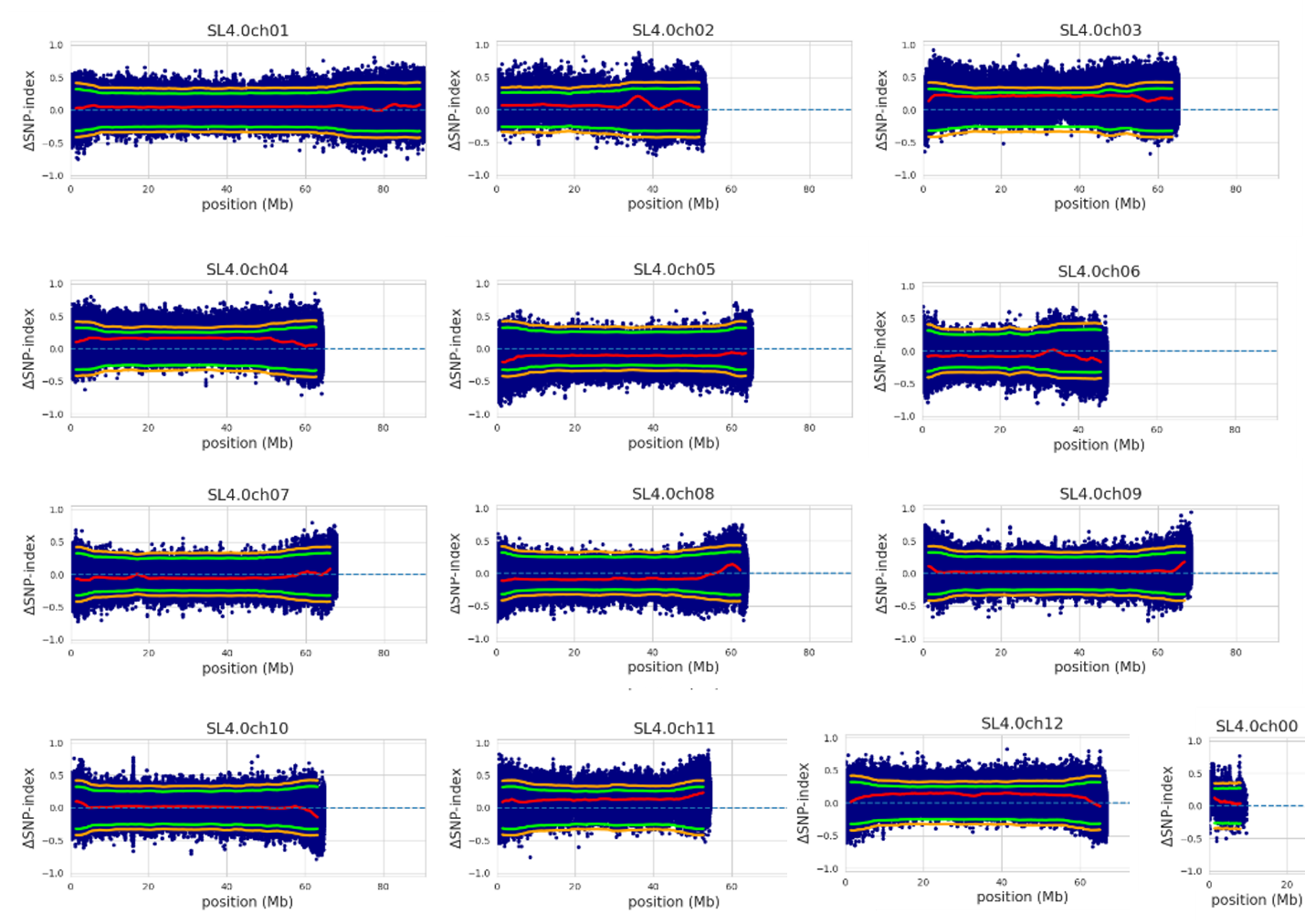
**

**Figure S5** QTL-seq plots consisting of a ∆(SNP-index) across all tomato chromosomes, as performed according to Sugihara et al. (2022). Two pools of F2 plants homozygous for the *S.p. Tm-1* allele were subjected to whole genome sequencing. The resistant pool consisted of 15 plants that showed no symptoms and were negative for ToBRFV viral coat protein, while the susceptible pool consisted of 32 plants that showed symptoms (DSI ≥ 1) and were positive for ToBRFV viral coat protein. The mean ∆(SNP-index) is shown as a red line, while statistical confidence intervals under the null hypothesis of no QTLs are shown in green (P < 0.05) and orange (P < 0.01).


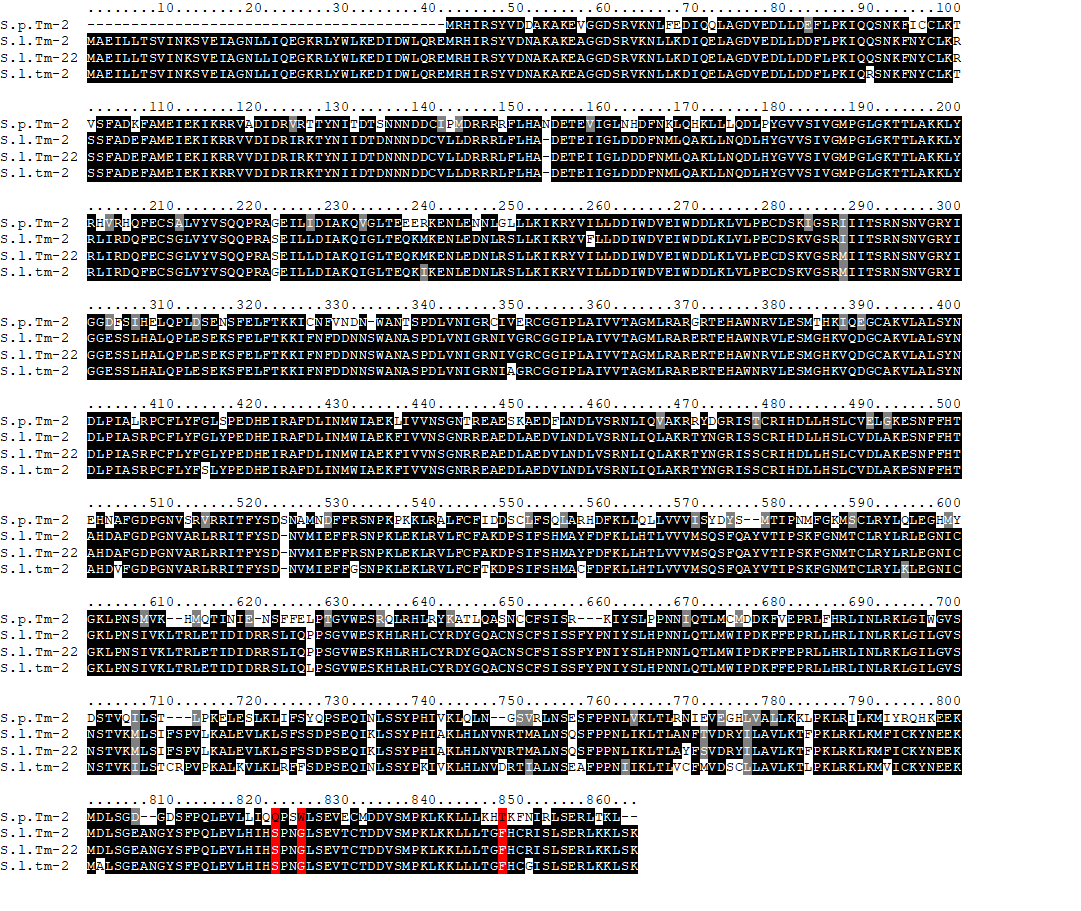


**Figure S6** Protein sequence alignment of S. pennellii LA 0716 Tm-2 (S.p. Tm-2, Sopen09g035210), the ToMV resistance Tm-2 (S.l. Tm-2, NCBI: AF536200), allelic variant Tm-2^2^ (S.l. Tm-22, NCBI: AF536201), and the ToMV susceptible tm-2 (S.l. tm-2, NCBI: AF536199). Amino acids highlighted in red (S822, G825 and F848) indicate the positions of the engineered substitutions in Tm-2^2^, which can confer resistance to ToBRFV (Lindbo 2022).


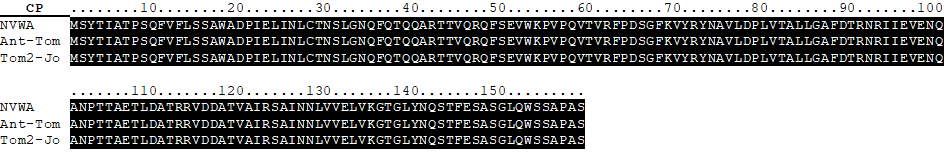


**Figure S7** Coat protein sequence alignment of ToBRFV isolates NVWA (NCBI: MN882011.1), CA18 (NCBI: MT002973.1) and Ant-TOM (NCBI: MT107885.1).


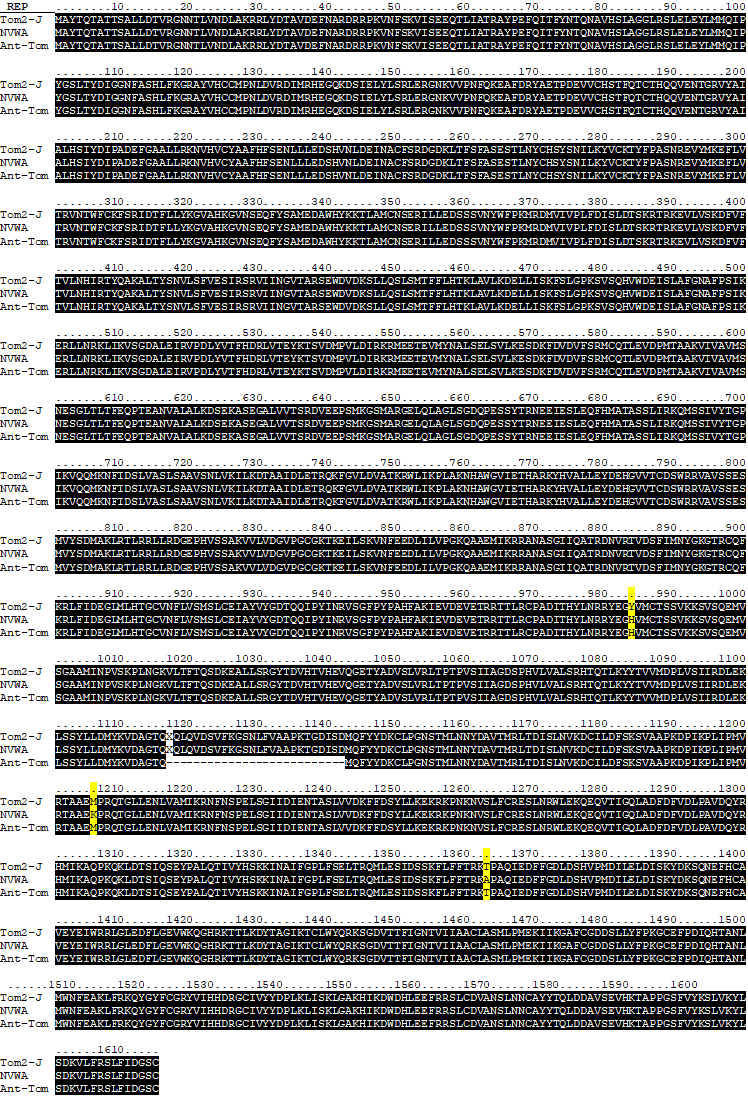


**Figure S8** Replication protein sequence alignment of ToBRFV isolates NVWA (NCBI: MN882011.1), CA18 (NCBI: MT002973.1) and Ant-TOM (NCBI: MT107885.1). The amino acid differences are highlighted with yellow colour.


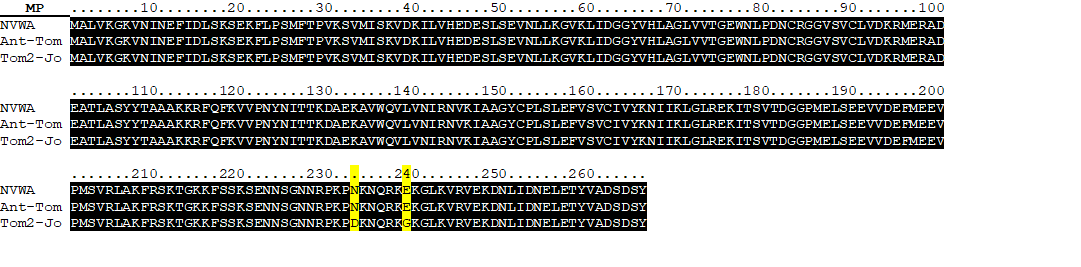


**Figure S9** Movement protein sequence alignment of ToBRFV isolates NVWA (NCBI: MN882011.1), CA18 (NCBI: MT002973.1) and Ant-TOM (NCBI: MT107885.1). The amino acid differences are highlighted with yellow colour.

**Table S1** Marker screening of individual F2 (LA 0716) plants of the R-pool and S-pool used for the BSA screening using Indel markers as described by Toal et al. (2016). Markers were scored as a (homozygous for the Moneymaker allele), h (heterozygous) or b (homozygous for the S. pennellii LA 0716 allele).
